# Psychopathological Dynamics of Obsessive-Compulsive Disorder in Visually Impaired Patients

**DOI:** 10.62641/aep.v53i6.1889

**Published:** 2025-12-17

**Authors:** Sergii Boltivets, Tymur Gonchar, Lyudmila Uralova, Oleksii Honchar, Yuliya Chelyadyn

**Affiliations:** ^1^Department of Scientific Support of Social Development of Youth, State Institute of Family and Youth Policy of Ukraine, 01001 Kyiv, Ukraine; ^2^Department of Psychiatry, Psychotherapy, and Medical Psychology, Shupyk National Healthcare University of Ukraine, 04112 Kyiv, Ukraine; ^3^Department of Radiology, Shupyk National Healthcare University of Ukraine, 04112 Kyiv, Ukraine

**Keywords:** low vision, perinatal injury, psychopathological structure, central nervous system, schizotypal disorder, obsessive-compulsive disorder, gestational age

## Abstract

**Background:**

Perinatal damage to the central nervous system affects both refractogenesis and the formation of central vision in young children. Directly acting on neuronal systems, brain damage can be the main cause of the pathogenesis of mental disorders that can manifest throughout life.

**Methods:**

The methods of neurosonography, computer tomography, magnetic resonance imaging, as well as clinical, psychodiagnostic, and statistical methods were used to study the peculiarities of the psychopathological structure of mental disorders in people with visual impairments due to perinatal pathology.

**Results:**

The severity of obsessive-compulsive disorder (OCD) symptoms showed that 59.4% of the F42 group had moderate symptoms, while 34.8% of the F21 group had serious symptoms (χ^2^ = 6.154, *p* = 0.013). Clinical anxiety was significantly more prevalent in the F42 group (53.1%) compared to the F21 group (13.0%) (χ^2^ = 27.774, *p* < 0.001), and clinical depression was also more frequent in the F42 group (24%) than in the F21 group (2.9%) (χ^2^ = 13.846, *p* < 0.001). A majority of F42 patients (60.4%) rated their quality of life as low, compared to 40.6% in the F21 group (χ^2^ = 6.324, *p* = 0.012). Regarding social support, 68.8% of F42 patients received support from family, compared to 49.3% in the F21 group (χ^2^ = 6.377, *p* = 0.012).

**Conclusions:**

The obtained data contribute to the development and implementation of the algorithm of differentiated medical and social rehabilitation of patients with visual impairments and mental disorders in order to improve their quality of life.

## Introduction

According to the World Health Organization, approximately 2.2 billion people worldwide have visual impairment [1, 2]. The relationship between visual impairment and psychiatric disorders was investigated among people aged 60 years and older in Tehran, Iran, by means of a population-based cross-sectional study. The study confirmed that as a result of vision impairment, people more often experienced functional problems, which, in turn, led to mental disorders [3]. Findings from 18 semi-structured interviews with patients diagnosed with eye disease resulting in vision loss suggest that mental health impacts include depression/low mood, anxiety, and stress-related visual impairment. Impacts on identity include coping with a limited life, concerns that visual impairments may define a person’s identity, and feelings of frustration at their own loss of function and the reactions of others to their disability. The future includes thoughts about long-term consequences, both negative and positive (e.g., maximizing experience given the vision that remains) [4].

A review of the existing literature on protective and risk factors for mental health in working-age adults with incidental total bilateral blindness and low vision includes publications that examine protective and risk factors for mental health in the study population. As has been pointed out in previous studies [5], the results of this study could be used as a starting point for more real-world research into the lives of working-age people who become totally blind on both sides by accident. Surgery or medication cannot correct gross violations of visual functions that constitute low vision, which is a rather broad concept. People with this condition can see much less clearly or have problems with their field of vision, such as blind spots or the edges of their field of vision getting smaller [6].

The role of perinatal hemorrhagic and hypoxic-ischemic cerebrovascular lesions in the development of vision pathology is significant, with their frequency ranging from 40% to 80–85%, inversely proportional to gestational age. These lesions negatively affect refractogenesis and central vision formation, with a growing prevalence of visually impaired children, particularly those with retinopathy caused by perinatal pathology. Acting on neural systems, such brain injuries can also lead to lifelong mental disorders that become more apparent during development [7].

The study of mental disorders arising from perinatal cerebrovascular damage in visually impaired individuals remains critical. Key issues include understanding their dynamics, clinical prognosis, and optimizing therapeutic and rehabilitation strategies tailored to clinical presentations. Current psychiatric rehabilitation often focuses on societal functioning and resource modification, but the lack of targeted programs addressing the specific needs of this population limits the effectiveness of treatment. Taking these problems into account will help with accurately diagnosing and classifying mental disorders and creating useful rehabilitation programs for people who are blind or have low vision [8].

Currently, among the approaches to psychiatric rehabilitation, more widely than the rehabilitation methods themselves, treatment methods are used, the purpose of which is to improve the functioning of the individual in society, programming skills, and modification of resources. The therapeutic potential of modern clinical practice is greatly limited by the lack of medical and social rehabilitation programs that take into account the clinical options, quality of life, and social functioning of people with mental illnesses in the population of people who are blind or have low vision because of damage to blood vessels during pregnancy [8].

Given the unresolved theoretical and practical challenges associated with mental disorders stemming from hemorrhagic and hypoxic-ischemic brain vessel lesions, further study of these issues remains crucial. Solving these problems will ensure the reliability of the psychopathological classification, the accuracy of the prognosis of these disorders, and help create rehabilitation programs to optimize the treatment process for patients with visual impairment [9].

Obsessive-compulsive disorder (OCD) is a disease that is primarily responsible for psychological as well as biological and social changes in the patient’s life. It affects the entire life of the individual, changing and destroying its micro- and macro-social ties. Today, there are studies devoted to the investigation of psychopathological manifestations of OCD, but the clinical typology of actual obsessive-compulsive (OC) symptoms remains inadequately researched and outlined within the framework of neurotic and sub-psychotic registers [10, 11, 12, 13].

The aim of this study was to examine the psychopathological structure and dynamics of mental disorders in individuals with visual impairments resulting from perinatal cerebrovascular lesions. The study focused on identifying clinical subtypes of OC symptoms, analyzing their psychological and social characteristics, and establishing a foundation for the development of differentiated therapeutic and rehabilitation strategies tailored to the specific needs of this patient group.

## Materials and Methods

### Study Design and Population

The present study was conducted over the years 1990 to 2019 as part of a comprehensive clinical follow-up investigation at the Institute of Nuclear Medicine and Radiology. Dynamic neurosonography was employed utilizing Microimager 1000 and 2000 machines (Ausonics, Sydney, New South Wales, Australia) and Aloca-360 (Aloka Co. Ltd., Tokyo, Tokyo Metropolis, Japan), while computed tomography was performed on CT9000NP (GE Healthcare, Milwaukee, WI, USA). Following standard procedures for pediatric research, MRI scans were done using Siemens Magneton P8 devices (Magneton P8, Siemens Healthineers, Erlangen, Bavaria, Germany) and Giroscan T5-NT devices from Philips (Philips Healthcare, Eindhoven, North Brabant, Netherlands).

The study extended to include a follow-up at the Department of Psychiatry, Psychotherapy, and Medical Psychology of the Shupyk National Medical Academy of Postgraduate Education, employing retrospective clinical analysis of medical records. These included outpatient cards and case histories, ensuring a thorough examination of changes in clinical presentation over time. The sample was constructed following the International Statistical Classification of Diseases and Related Health Problems, 10th Revision (ICD-10) [14], with participants stratified into two primary groups.

The neurotic group (F42) was made up of 96 visually impaired people (30 men and 66 women) who had the neurotic type of OCD. They had conditions like congenital optic nerve pathology, amblyopia, retinal disorders, and nystagmus. In contrast, the sub-psychotic group (F21) included 69 individuals (45 men and 24 women) with schizotypal disorder, characterized by sub-psychotic manifestations. The age of participants ranged from 16 to 29 years, and individuals with underlying endogenous pathologies were excluded from both groups.

Inclusion criteria for participants involved a confirmed diagnosis of OCD (F42 group) or schizotypal disorder (F21 group) based on ICD-10 guidelines, alongside documented visual impairments for the F42 group. Patients were required to be between the ages of 16 and 29 and to have sufficient cognitive capacity to participate in psychometric evaluations. Exclusion criteria eliminated individuals with comorbid endogenous disorders, severe intellectual disabilities, or active psychotic episodes, ensuring homogeneity within the study population and validity of the comparisons.

### Psychodiagnostic and Statistical Methods

The study’s clinical and psychopathological approach involved structured examinations to analyze the structure and dynamics of the disorders under investigation. The following psychodiagnostic tools were used: the Yale-Brown Obsessive-Compulsive Scale (Y-BOCS) [15] for assessing OCD severity, the Hospital Anxiety and Depression Scale (HADS) [16] for evaluating anxiety and depression, the Multidimensional Inventory of Hypochondriacal Traits (MIHT) [17], the Quality-of-Life Scale, the Multidimensional Scale of Perception of Social Support (MSPSS) [18], and the Life Style Index (LSI) [19]. These tools were applied consistently across both patient groups, enabling a comparative analysis of symptomatology, emotional states, quality of life, and coping strategies.

Statistical analyses employed correlation and factor analysis to assess relationships among variables and elucidate latent patterns within the data. Analyses were performed using SPSS 16.0 (Armonk, NY, USA) and Microsoft Excel from the Office 2003 suite, ensuring methodological robustness. The chi-square test was used for comparing categorical data. Statistical significance was determined using *p*-values. All results were systematically presented in tabular format, reflecting the rigor of the statistical approach.

Uniform statistical methodologies were applied to analyze differences between groups. Descriptive statistics were used to summarize the data, followed by inferential statistical tests to evaluate group differences. Specifically, *p*-values were calculated to assess the statistical significance of variations in OCD severity, levels of anxiety and depression, quality of life, perceived social support, and coping strategies. These methods ensured a comprehensive and objective evaluation of the study findings while maintaining methodological consistency across all analyses.

The statistical analysis relied on correlation and factor analysis techniques to explore interrelationships and identify underlying dimensions within the data. Correlation analysis was applied to assess the strength and direction of relationships among variables such as OCD severity, anxiety, depression, and coping strategies. The oblique quartimax rotation method was used for factor analysis to find the most important factors that affected symptoms and coping behaviors. Descriptive and inferential methods were integrated to provide a holistic interpretation of the results, focusing on both individual and group-level patterns. These methodologies ensured the reliability of the conclusions drawn and informed targeted therapeutic interventions for each patient group.

## Results

### Symptom Severity and Psychological Profiles

The study population consisted of 165 participants divided into two diagnostic groups: individuals with OCD of the neurotic type (F42) and those with schizotypal disorder of the sub-psychotic type (F21). The demographic composition included both male and female patients, with the F42 group having a higher proportion of women, while men were predominant in the F21 group. The participants’ ages ranged from 16 to 29 years, representing a developmental period characterized by significant psychological and social transitions. The neurotic group (F42) stood out because many of its members had both birth defects and acquired vision problems, like amblyopia and retinal pathologies. These are conditions that can make the mental burden of OCD even heavier. On the other hand, the sub-psychotic group (F21) showed signs of schizotypal disorder, such as sub-threshold psychotic symptoms and social withdrawal. However, these people did not have any underlying pathology, which ensured the accuracy of the comparison. Both groups shared a commonality in experiencing disruptions to their social functioning, albeit with distinct manifestations. The F42 group often showed a higher sensitivity to outside stressors and a strong tendency toward behavior driven by anxiety. On the other hand, the F21 group showed a more noticeable flattening of emotions and less social engagement. These clinical traits helped us understand the different types of psychopathology and symptoms in each group. They also gave us useful information about how psychiatric and neurodevelopmental factors interact.

Analysis was conducted on the specificity of features of psychopathological manifestations and the typology of OC symptoms in patients within neurotic and sub-psychotic registers prior to treatment [8, 9]. The severity of OC symptoms during the course of the disease yielded the following indicators (Table 1).

**Table 1.** **The OCD symptoms severity in neurotic and sub-psychotic patients, Y-BOCS scores**.

Severity of OCD symptoms	F42 (n = 96)	F21 (n = 69)	Сhi-square value (χ^2^)	*p*-value
Slight	18 (18.8%)	2 (2.9%)	9.456	0.002
Moderate	57 (59.4%)	32 (46.4%)	2.713	0.099
Serious	17 (17.7%)	24 (34.8%)	6.154	0.013
Very serious	4 (4.2%)	11 (15.9%)	7.283	0.007

OCD, obsessive-compulsive disorder; Y-BOCS, Yale-Brown Obsessive-Compulsive Scale.

Table 1 presents a comparison of OCDsymptom severity between the neurotic (F42) and subpsychotic (F21) groups using the Y-BOCS. Results showed that the majority of patients in the F42 group experienced moderate symptoms (59.4%), with a smaller proportion reporting severe (17.7%) or very severe symptoms (4.2%). In contrast, the F21 group had a higher percentage of serious (34.8%) and very serious (15.9%) symptoms compared to the F42 group, with 46.4% of patients reporting moderate symptoms. Statistical comparison revealed significant differences between the two groups in the proportion of patients with slight symptoms (χ^2^ = 9.456, *p* = 0.002), serious symptoms (χ^2^ = 6.154, *p* = 0.013), and very serious symptoms (χ^2^ = 7.283, *p* = 0.007). However, there was no significant difference in the proportion of patients with moderate symptoms (χ^2^ = 2.713, *p* = 0.099). The findings suggest that patients in the F21 group may have more severe OCD symptoms than patients in the F42 group, indicating the need for differentiated therapeutic approaches depending on the severity of symptoms. Investigating the severity of anxiety and depression in the neurotic and sub-psychotic groups, the following was found (Table 2).

**Table 2.** **Evidence of anxiety and depression, HADS scores**.

Evidence	F42 (n = 96)	F21 (n = 69)	Сhi-square value (χ^2^)	*p*-value
Subclinical anxiety	34 (26.4%)	28 (41.2%)	0.692	0.406
Subclinical depression	21 (16.3%)	29 (42.7%)	7.720	0.005
Clinical anxiety	51 (39.5%)	9 (13.2%)	27.774	<0.001
Clinical depression	23 (17.8%)	2 (2.9%)	13.846	<0.001

HADS, Hospital Anxiety and Depression Scale.

The comparison of anxiety and depression levels between the neurotic (F42) and sub-psychotic (F21) groups shows that clinical anxiety was significantly more prevalent in the F42 group, with 53.1% of patients experiencing clinical anxiety compared to just 13.2% in the F21 group (χ^2^ = 27.774, *p* < 0.001). Similarly, clinical depression was significantly higher in the F42 group (24%) than in the F21 group (2.9%) (χ^2^ = 13.846, *p* < 0.001). In contrast, subclinical anxiety was observed in 35.4% of F42 patients and 40.6% of F21 patients, with no statistically significant difference (χ^2^ = 0.692, *p* = 0.406). However, subclinical depression was significantly more common in the F21 group (42.7%) compared to the F42 group (21.9%) (χ^2^= 7.720, *p* = 0.005).

These results underscore the greater psychological burden in the F42 group, where anxiety and depression are more pronounced, while the F21 group shows a tendency toward less severe forms of these symptoms. This suggests that therapeutic interventions for anxiety and depression may need to be more intensive for patients in the neurotic group. It is the feature that can further determine the strategies of therapeutic measure assignment. The assessment of patients’ quality of life in the F42 and F21 groups was as follows (Table 3).

**Table 3.** **Quality of Life Scale scores**.

Level of Life	F42 (n = 96)	F21 (n = 69)	Сhi-square value (χ^2^)	*p*-value
Medium	22 (22.9%)	38 (55.1%)	18.021	<0.001
Low	58 (60.4%)	28 (40.6%)	6.324	0.012
Very low	16 (16.7%)	3 (4.3%)	5.980	0.014

The results indicate a significant difference in perceived quality of life between the two groups. A greater proportion of patients in the F42 group reported a low quality of life (60.4%) compared to 40.6% in the F21 group, and this difference was statistically significant (χ^2^ = 6.324, *p* = 0.012). Conversely, 55.1% of patients in the F21 group rated their quality of life as medium, while only 22.9% of patients in the F42 group reported the same, with this difference being highly significant (χ^2^ = 18.021, *p* < 0.001). Additionally, very low quality of life was more frequently reported in the F42 group (16.7%) compared to the F21 group (4.3%) (χ^2^ = 5.980, *p* = 0.014).

These findings suggest that patients in the F42 group have a significantly lower quality of life, which may be related to the more severe psychological symptoms, such as anxiety and depression, observed in this group. In contrast, the F21 group appears to have a more stable perception of quality of life, which may be associated with relatively less severe psychological distress. These differences emphasize the need for targeted intervention to improve quality of life in the neurotic group.

### Social Support and Defense Mechanisms

A very low standard of living may indicate a subobjective (His-dystonic) perception of the disease and the inability to function well in the macro- and micro-societies. Thus, the peculiarities of the perception of social support in the groups of patients in the neurotic and endogenous registers were studied (Table 4).

**Table 4.** **Social support depending on the register of disease, MSPSS scores**.

Social support type	F42 (n = 96)	F21 (n = 69)	Сhi-square value (χ^2^)	*p*-value
Family	66 (68.8%)	34 (49.3%)	6.377	0.012
Colleagues at work	6 (6.3%)	1 (1.4%)	2.254	0.133
Friends	26 (27.1%)	4 (5.8%)	12.228	<0.001
Significant others	18 (18.8%)	21 (30.4%)	2.941	0.086
Public organizations	4 (4.2%)	2 (2.9%)	0.183	0.668

MSPSS, Multidimensional Scale of Perception of Social Support.

As can be seen from the data presented, patients from the F42 group have 68.8% support in the family and 27.1% support by friends. The majority of patients in the F21 group (49.3%) can also find support in the family. Significantly more support from the family (χ^2^ = 6.377, *p* = 0.012) and friends (χ^2^ = 12.228, *p* < 0.001) is experienced by patients in the F42 group comparing to the F21 group. However, no significant differences were observed in support from colleagues at work (χ^2^ = 2.254, *p* = 0.133), significant others (χ^2^ = 2.941, *p* = 0.086), or public organizations (χ^2^ = 0.183, *p* = 0.668) between the two groups.

Studying the mechanisms of psychological protection for the individual with OC symptoms of neurotic and sub-psychotic types, it was possible to construct a protective structure profile in groups F42 and F21 (Table 5).

**Table 5.** **Psychological protection mechanisms according to the Lifestyle Index scores**.

Psychological protections	F42 (n = 96)	F21 (n = 69)	Сhi-square value (χ^2^)	*p*-value
Suppression	72 (75.0%)	42 (60.9%)	3.754	0.053
Regression	31 (32.3%)	40 (58.0%)	10.799	0.001
Substitution	3 (3.1%)	43 (62.3%)	70.047	<0.001
Negation	8 (8.3%)	18 (26.1%)	9.286	0.002
Projection	20 (20.8%)	13 (18.8%)	0.099	0.752
Compensation	16 (16.7%)	4 (5.8%)	4.441	0.035
Reactive formation (hypercompensation)	66 (68.8%)	26 (37.7%)	16.475	<0.001
Rationalization (intellectualization)	57 (59.4%)	12 (17.4%)	29.130	<0.001
Total intensity of protective mechanisms	72 (75.0%)	17 (24.6%)	40.986	<0.001

As shown in the table, patients in the F42 group most often utilized the following mechanisms of protection: suppression (75.0%), reactive formation (68.8%), and rationalization (59.4%). In contrast, patients in the F21 group with sub-psychotic type employed suppression (60.9%), regression (58.0%), substitution (62.3%), and significantly less hypercompensation (37.7%).

When comparing the F42 group to the F21 group, a significantly higher number of patients in the F42 group used protective mechanisms such as compensation (χ^2^ = 4.441, *p* = 0.035), reactive formation (hypercompensation) (χ^2^ = 16.475, *p* < 0.001), and rationalization (intellectualization) (χ^2^ = 29.130, *p* < 0.001). Conversely, patients in the F21 group were significantly more likely to utilize regression (χ^2^ = 10.799, *p* = 0.001), substitution (χ^2^ = 70.047, *p* < 0.001), and negation (χ^2^ = 9.286, *p* = 0.002) in relation to the F42 group. Projection (χ^2^ = 0.099, *p* = 0.752) did not show significant differences between the groups.

In the neurotic group (F42), the overall intensity of protective mechanisms is 75.0%, reflecting existing but unresolved external and internal conflicts. In the sub-psychotic group (F21), the intensity is 24.6%, significantly lower than 50%. A significant role in the research was attributed to studying the peculiarities of choosing coping strategies in patients from the neurotic group (F42) and the sub-psychotic group (F21), where a shift from a nosocentric to an adaptive psychotic paradigm occurred (Table 6).

**Table 6.** **Specifics of coping strategy choosing (by the methodology for psychological diagnostics of coping)**.

Coping strategy	F42 (n = 96)	F21 (n = 69)	Сhi-square value (χ^2^)	*p*-value
Confrontation	4 (4.2%)	1 (1.4%)	1.014	0.314
Distancing	52 (54.2%)	13 (18.8%)	20.954	<0.001
Self-control	65 (67.7%)	51 (73.9%)	0.741	0.389
Search for social support	43 (44.8%)	12 (17.4%)	13.563	<0.001
Acceptance of responsibility	46 (47.9%)	6 (8.7%)	28.600	<0.001
Escape-avoidance	43 (44.8%)	38 (55.1%)	1.697	0.193
Planning for problem solving	43 (44.8%)	11 (15.9%)	15.134	<0.001
Positive revaluation	7 (7.3%)	1 (1.4%)	2.973	0.085

The study of coping strategies examined how patients interact with their environment through the lens of OC symptoms within the neurotic and sub-psychotic modes. This analysis shifted the focus from unconscious intra-psychic protective mechanisms to the exploration of active and purposeful behaviors related to OCD, termed inter-psychic mechanisms. In the F21 group, typical coping strategies included self-control (73.9%) and escape-avoidance (55.1%). In contrast, the F42 group exhibited characteristic coping strategies of self-control (67.7%) and escape-avoidance (44.8%). Notably, neurotic patients (F42) were significantly more likely than sub-psychotic patients (F21) to utilize coping strategies such as distancing (χ^2^ = 20.954, *p* < 0.001), seeking social support (χ^2^ = 13.563, *p* < 0.001), accepting responsibility (χ^2^ = 28.600, *p* < 0.001), and planning for problem-solving (χ^2^ = 15.134, *p* < 0.001).

### Factor Analysis and Symptom Typology

A correlation matrix was made from each person’s scores on the Y-BOCS symptoms checklist during the first part of the OC symptoms typology study. The primary data array was constructed from a list of patient symptoms derived from Y-BOCS assessments. The factor characteristics were used to figure out the correlation matrix, and the oblique quartimax rotation method was then used for statistical analysis. This analysis resulted in the identification of main components (factors) and their corresponding weights expressed in percentages (Table 7).

**Table 7.** **Main components weights (dispersions)**.

Main component (factor)	Eigenvalue	Dispersion (%)	Cumulative weight of factors
1	1.571	27.15	27.15
2	1.326	22.93	50.08
3	1.125	19.45	69.53
4	0.942	16.29	85.82
5	0.313	5.41	91.23
6	0.198	3.42	94.66
7	0.095	1.64	96.30
8	0.081	1.40	97.70
9	0.052	0.90	98.60
10	0.033	0.57	99.17
11	0.021	0.36	99.53
12	0.020	0.35	99.88
13	0.004	0.07	99.95
14	0.003	0.05	100.00

The findings show that the first four factors make up 85.8% of the weights of all the major components. Because of this, the study focused on these four factors and how to interpret them. The next step involved analyzing the significance of individual symptoms in the formation of factor loads, which were presented as correlation coefficients.

The methodological foundation of this analysis was the quartimax procedure, which entailed the rotation of factors (matrix rotation). This method lets each variable (in this case, a certain symptom) have a strong relationship with one factor. This makes it easier to distribute factors more accurately based on groups of specific symptoms that have strong relationships with each chosen factor. In the scientific literature, this technique is also referred to as the implementation of the totality of factor decisions principle (L. Thurstone).

As we investigated two groups of patients with OC symptoms of neurotic and sub-psychotic type, we conducted a similar factor analysis within each group. The purpose of this analysis was to determine the specificity of the distribution of factors within each group. The results of the analysis are shown in the Tables 8,9.

**Table 8.** **Factor loads obtained as a result of quartimax rotation of the Y-BOCS scoring scale for the group of neurotic patients (F42)**.

Symptoms	Factor loads (n = 96)			
	Incompleteness	Danger	Ambivalence	Accumulation
Symmetry and order obsessions	0.526	0.385	0.218	0.143
Contamination obsessions	0.160	0.781	0.054	–0.104
Aggressive thoughts	0.022	0.228	0.686	0.017
Obsessions of hypochondriacal content	–0.366	0.589	0.019	0.099
Obsessions with sexual content	0.165	0.072	0.814	–0.086
Obsessions with religious content	–0.068	0.646	0.123	–0.190
Obsessions with dysmorphophobical content	–0.011	0.053	0.813	–0.030
Other obsessions	0.044	–0.013	0.068	0.325
Compulsions of symmetry and order	0.895	0.217	0.084	–0.015
Rituals of repetition	0.637	0.377	0.087	0.064
Compulsions of cleansing	0.252	0.511	–0.335	–0.153
Compulsive checks	0.195	0.802	–0.009	0.101
Compulsive neurotic excoriation	0.083	–0.005	0.532	0.042
Compulsions of gathering and collecting	0.102	0.188	–0.140	0.349

Y-BOCS, Yale-Brown Obsessive-Compulsive Scale.

**Table 9.** **Factor loads obtained as a result of quartimax rotation of the Y-BOCS scoring scale for the group of neurotic patients (F21)**.

Symptoms	Factor loads (n = 69)			
	Incompleteness	Danger	Ambivalence	Accumulation
Symmetry and order obsessions	0.270	0.372	0.225	0.268
Contamination obsessions	0.074	0.692	0.055	–0.226
Aggressive thoughts	0.011	0.212	0.735	0.033
Obsessions of hypochondriacal content	–0.158	0.581	0.019	0.230
Obsessions with sexual content	0.076	0.081	0.721	–0.187
Obsessions with religious content	–0.027	0.514	0.155	–0.481
Obsessions with dysmorphophobical content	–0.005	0.058	0.753	–0.068
Other obsessions	0.018	–0.009	0.092	0.797
Compulsions of symmetry and order	0.343	0.209	0.087	–0.041
Rituals of repetition	0.307	0.326	0.101	0.132
Compulsions of cleansing	0.111	0.569	–0.301	–0.347
Compulsive checks	0.081	0.874	–0.008	0.244
Compulsive neurotic excoriation	0.037	–0.005	0.534	0.094
Compulsions of gathering and collecting	0.047	0.193	–0.137	0.750

Y-BOCS, Yale-Brown Obsessive-Compulsive Scale.

As can be seen from the data for neurotic patients, the first three factors (types) that remain significant are—type 1—obsession of incompleteness, type 2—obsession of danger, type 3—ambivalent obsession (forbidden motives). But for neurotic patients, correlations with the factor (type 4) disappear (the obsession of accumulation).

For the group of sub-psychotic patients, three factors (types) remain significant: type 2—obsession with danger, type 3—ambivalent obsession (forbidden motives), and type 4—obsession with accumulation. In this case, for sub-psychotic patients, correlations with the factor (type) 1 (an obsession of incompleteness) disappear.

Consequently, type 1 and type 4 can be defined as special. For neurotic patients—type 1 (an obsession of incompleteness) and for sub-psychotic ones—type 4 (an obsession of accumulation) are the most important. Based on the results obtained, we distributed the neurotic and sub-psychotic patients according to the priority of symptoms between the types, which in the future would receive specific therapy. Accordingly, specific therapy was assigned to each type of patients in F42 and F21 groups.

The study revealed significant differences in the psychopathological characteristics and symptomatology between the neurotic (F42) and sub-psychotic (F21) groups. Among the F42 group, 59.4% of patients exhibited moderate OCD symptoms, while 18.8% experienced slight symptoms and 17.7% had serious symptoms, compared to the F21 group, where 46.4% showed moderate symptoms and 34.8% had serious symptoms (χ^2^ = 6.154, *p* = 0.013). Anxiety and depression were more pronounced in the F42 group, with 53.1% displaying clinical anxiety and 17.8% clinical depression, in contrast to 13.2% and 2.9%, respectively, in the F21 group (χ^2^ = 27.774, *p* < 0.001). Quality of life assessments showed that 60.4% of the F42 group reported a low standard of living, compared to 40.6% in the F21 group (χ^2^ = 6.324, *p* = 0.012), where 55.1% of the F21 group rated their quality of life as average, compared to 22.9% in the F42 group (χ^2^ = 18.021, *p* < 0.001). Regarding social support, 68.8% of F42 patients received family support versus 49.3% in the F21 group (χ^2^ = 6.377, *p* = 0.012). Support from friends was significantly higher in the F42 group (27.1%) than in the F21 group (5.8%) (χ^2^ = 12.228, *p* < 0.001).

Mechanisms of psychological defense showed that suppression (75.0%), reactive formation (68.8%), and rationalization (59.4%) were predominant in the F42 group, whereas regression (58.0%) and substitution (62.3%) were more frequent in the F21 group (*p* < 0.001). Finally, factor analysis identified distinct clinical subtypes of OCD symptoms, including incompleteness (49 cases in F42, χ^2^ = 50.100, *p* < 0.001), danger (26 cases in F42 and 21 in F21, χ^2^ = 0.181, *p* = 0.670), ambivalence (21 cases in F42 and 30 in F21, χ^2^ = 8.773, *p* = 0.003), and accumulation (18 cases in F21 only, χ^2^ = 27.949, *p* < 0.001), providing a foundation for targeted therapeutic interventions.

## Discussion

The findings of this study align with existing literature highlighting the intricate relationship between mental disorders and visual impairments, while also providing new insights into the psychopathological dynamics of OCD in visually impaired patients. Hashemi *et al*. [3] emphasize the strong association between visual impairment and the prevalence of mental health disorders, illustrating that vision loss is not merely a physical condition but one deeply intertwined with psychological outcomes. This link is clear from the study’s results, which showed that people in the neurotic OCD group (F42) had much higher levels of anxiety and depression than people in the sub-psychotic group (F21). This suggests that losing your sight may make your OCD symptoms worse.

The psychological impact of vision loss, as explored by Boagey *et al*. [4], underscores the profound influence of visual deficits on emotional well-being and social functioning. Their findings align with the current study’s results, which demonstrated lower quality of life scores and reduced perceived social support among patients with OCD and visual impairments. The correlation between these factors may stem from the social isolation and reduced autonomy that often accompany vision loss, further exacerbating OCD symptoms, particularly in the neurotic group. Dike *et al*. [5] provide a broader context by identifying protective and risk factors for mental health in individuals with severe visual impairments. They highlight the importance of resilience-building strategies and social support systems. In the same way, this study showed that the neurotic group had more support from family and friends than the sub-psychotic group. This suggests that better social networks may lessen the severity of mental health disorders in visually impaired people. However, the discrepancies in coping mechanisms between the two groups underline the need for tailored interventions addressing specific psychopathological profiles.

From the point of view of neurodevelopment, Boonstra *et al*. [20] talk about the multidisciplinary guidelines for cerebral visual impairment (CVI), stressing how important it is to diagnose and treat CVI as soon as possible. This aligns with findings by Rees *et al*. [21], who documented the long-term neurodevelopmental consequences of perinatal brain injuries. Both sources provide a foundation for understanding the developmental trajectories observed in the current study’s participants. The study found that perinatal hypoxic-ischemic and hemorrhagic brain lesions can lead to both vision problems and mental health problems. This supports Chokron and Dutton’s [22] claim that vision problems in the brain can cause problems with cognitive and neurodevelopmental development. Further, the neurological basis of OCD in visually impaired patients can be contextualized by the work of Oh *et al*. [23], who identified abnormal brain activation patterns in OCD patients. The combined data on the distribution of neurotic and subpsychotic patients by the prevalence of specific symptoms are presented in Table 10. Their findings suggest that disruptions in visuospatial processing may underlie some OCD symptoms. This resonates with the current study’s observations of distinct symptom typologies in the neurotic and sub-psychotic groups, emphasizing the need to consider neurophysiological factors in therapeutic planning. The study by Mani *et al*. [24] on episodic memory deficits in OCD patients also adds depth to the understanding of the cognitive challenges faced by visually impaired individuals with OCD. While this study was mostly about psychopathological dynamics, the cognitive impairments pointed out by Mani *et al*. [24] may help explain why the neurotic group had more anxiety and depression, possibly because of problems with memory and executive functioning.

**Table 10.** **Distribution of neurotic and sub-psychotic patients according to the prevalence of specific symptoms**.

Type	F42 (n = 96)	F21 (n = 69)	Сhi-square value (χ^2^)	*p*-value
Incompleteness	49	0	50.100	<0.001
Danger	26	21	0.181	0.670
Ambivalence	21	30	8.773	0.003
Accumulation	0	18	27.949	<0.001

Finally, Morein-Zamir *et al*. [25] investigated attentional biases in OCD and noted post-attentional processing abnormalities. Although this study did not directly assess attentional mechanisms, the observed differences in coping strategies and psychological defenses between groups may reflect underlying attentional and cognitive processing differences. For example, the neurotic group’s greater use of rationalization and hypercompensation may mean that they are more aware and sensitive to their condition, while the sub-psychotic group’s preference for denial and substitution may mean that they are less aware and sensitive to their condition.

Taken together, these findings contribute to a growing body of literature that emphasizes the multifactorial nature of OCD in visually impaired individuals. The interplay between visual impairments, psychopathological factors, and social support systems underscores the need for comprehensive, interdisciplinary approaches to treatment. To help this group of people with their unique problems, future research should look into how to combine cognitive and behavioral therapies with vision rehabilitation programs. This holistic perspective will not only improve therapeutic outcomes but also enhance the quality of life and social functioning for visually impaired individuals with OCD.

## Conclusions

The study shows that perinatal cerebrovascular lesions play a big role in the development of visual impairments and the mental disorders that go along with them. It also shows how common four different types of obsessive-compulsive disorders are: incompleteness, danger, ambivalence, and accumulation. To improve the quality of life of people who are blind or have low vision, these results show how important it is to have a medical and social rehabilitation program that is tailored to their specific clinical and psychopathological needs.

## Availability of Data and Materials

All data generated or analyzed during this study are included in this published article. Individual survey data are stored on paper at the Scientific and Research Center “Olexandr Yaremenko Institute of Family and Youth Policy ” of the Mykhailo Dragomanov Ukrainian State University Educational and Scientific Institute of Youth and Social Policy at the address: Ukraine, 04119, Kyiv, Illenko St., 46 and can be provided upon written motivated request in the amount corresponding to the written consent of each surveyed person.
